# Identifying the mentorship needs among faculty in a large department of psychiatry- support for the creation of a formal mentorship program

**DOI:** 10.1186/s12909-024-06629-y

**Published:** 2025-01-11

**Authors:** Mary Jane Esplen, Lisa M. Fiksenbaum, Elizabeth Lin, Shaheen A. Darani, John Teshima, Simone N. Vigod, Nicole Kozloff, Peter Szatmari, Krista L. Lanctôt, Certina Ho, Ivan Silver, Sophie Soklaridis, Jiahui Wong

**Affiliations:** 1 University of Toronto, Toronto, ON Canada; 2 Women’s College Hospital, Toronto, ON Canada; 3 Centre for Addiction and Mental Health, Toronto, ON Canada; 4 Sunnybrook Health Sciences Centre, Toronto, ON Canada; 5 de Souza Institute, University Health Network, Toronto, ON Canada

**Keywords:** Mentorship, Psychiatry Department, Priorities in mentorship; academic medicine, Inequities in mentorship

## Abstract

**Background:**

Study aims were to assess the current state and needs of faculty to inform the design of a formal mentorship program in a large academic Department of Psychiatry.

**Methods:**

A 57- item self-administered online survey questionnaire was distributed to all faculty members.

**Results:**

225 faculty members completed the survey (24%). 68% of respondents had a mentor and reported high satisfaction (mean = 4.3, SD = 1.05) (range 1 to 5). Among those respondents lacking access to mentorship, 65% expressed interest. Open-ended questions indicated that international medical graduates, faculty identifying as minority, women and clinician teachers may lack access to mentorship. PhD faculty felt disadvantaged compared to MD faculty in gaining first authorship (*M*_Non−MD_=1.64 ± 0.79 vs. M_MD_=1.36 ± 0.67; *t* = 2.51, *p* = .013); reported more authorship disputes (*M*_Non−MD_ =1.99 ± 0.91 vs. M_MD_ =1.66 ± 0.76; *t* = 2.63 *p* = .009) and experienced questionable scientific integrity concerning colleagues (*M*_Non−MD_ =2.01 ± 0.92 vs. M_MD_ =1.70 ± 0.81; *t* = 2.42 *p* = .017). For both MD and PhD faculty, women were significantly more likely to experience authorship disputes (χ^2^(2) = 8.67, *p* = .013). The department was perceived as treating faculty with respect (72% agreed) with 54% agreeing that it embraces diversity (54%). Identified benefits to mentorship included receiving advice about academic promotion, opportunities for career advancement, advocacy, and advice as a researcher, teacher or clinician. Only 26% of mentors received formal training to support their role; 59% expressed interest in education. Respondents supported a more formal, accessible, inclusive program, with training, tools, and a matching strategy based on mentee preferences.

**Conclusions:**

Challenges and inequities were identified with the department’s current ad hoc approach to mentorship. A limitation of the study was the response rate, while similar to response rates of other physician surveys, raises the potential for response bias. In comparing study participants to the department, the sample appeared to provide a fair representation. The study has implications for identifying the need and design of more formal mentorship programs in academic medicine.

**Supplementary Information:**

The online version contains supplementary material available at 10.1186/s12909-024-06629-y.

Mentorship has a critical role in the development of academic physicians and scientists. It has been linked to several outcomes, including academic productivity and promotion, career satisfaction, and faculty retention [1–4]. Mentorship generally involves a senior experienced faculty member providing ongoing professional support to a junior faculty member [5], with the roles of mentors typically falling into the categories of career and psychosocial functions. Career-related functions promote professional advancement and skill development and include sponsorship, exposure, visibility, coaching, and provision of opportunities [6]. Psychosocial mentoring functions involve encouragement, role modeling, and counseling [6, 7]. Socialization into a new role is important for all junior faculty to successfully integrate into the social culture of the work environment.

The most common mentorship model is the dyad approach, however peer group mentorship has been used to address the costs associated with 1:1 mentorship programs [1, 8]. Important factors associated with successful mentorship programs are having clear goals, curriculum training to support relationship development between mentors/ mentees, access to diverse relationships and opportunities for networking [5]. Key challenges to uptake and sustainability of programs include lack of protected time for faculty and costs, logistics of setting up meetings/ or groups, physical distance (to attend mentorship meetings), and mentees’ perceptions of mentoring relationships, for example, being superficial or exploitive [1, 4, 9]. Personal factors or relational difficulties, lack of cultural awareness and institutional /structural issues (e.g. not valuing mentorship nor providing incentives) are also known barriers [2, 4, 9]. Mission statements and contracts or other ways to show commitment appear to enforce accountability and support sustainability [1]. While success with programs may be associated with salary support to compensate for training or the time involved [5, 6, 8], many programs are unable to provide support for faculty member time to participate. However, having dedicated support staff or other structures, such as expectations, goals, or a requirement to participate may facilitate success of programs [5].

Elevating mentorship to the level of a strategic priority has been emphasized by Choi et al. [10] as key to promoting access to high-quality mentorship in academic medicine. The Department of Psychiatry at the Temerty Faculty of Medicine, University of Toronto (Toronto, Ontario, Canada) (hereafter referred to as the “Department”), comprises nearly 1,000 academic faculty situated across 20 academic health and community sites in Toronto. Physician faculty members generally hold non-tenure clinical positions and participate in scholarly activities, although roles may differ (e.g. clinician scientist; clinician teacher). The Department also includes PhD Scientists or PhD-level clinical faculty with teaching responsibilities. While a pilot program in the Department offered mentoring to residents [11], mentorship for faculty was provided informally, on an ad hoc basis. Departmental culture was one where participation in scholarly activities was expected, including teaching. Mentorship was often encouraged; however, the department did not have a formal requirement for faculty to have a mentor nor to provide mentoring. There also was no formal system to assist a junior faculty member to find a mentor. Financial support towards mentorship was not provided. A formal academic mentorship program was named a key deliverable in the Department’s 2020–2026 strategic plan. Faculty needs and the proportion of those without mentors were unknown.

In 2020, a needs assessment was conducted aiming to assess the current state and needs of faculty and identify important components to consider in the design and implementation of a more formalized mentorship program. This paper reports on its findings and highlights disparities and challenges that can arise in the absence of a more systematic approach to providing mentorship.

## Methods

A needs assessment is a set of tools and processes used to collect information about a target audience’s needs. Needs can include gaps in the target audience’s knowledge, skills, performance, and/or health outcomes, requiring improvement [12]. Typically, a needs assessment strategy uses multiple sources and/or methods to collect information about the target audience’s needs: both perceived and unperceived. Unperceived needs pertain to those that members of the target audience may not be aware of (target audience may not report these gaps) and involve methods for their identification, along with approaches that obtain information on perceived needs.

For this needs assessment, we aimed to answer a series of questions that were deemed essential to the successful development and implementation of a new mentorship program. Specifically, the needs assessment goals were to: (1) assess the mentorship needs of faculty members; (2) review the current state of mentorship opportunities and experiences; and (3) review the departmental context with an emphasis on departmental resources and potential barriers.

As a first step, we consulted the literature in academic medicine to identify specific aspects of need related to mentorship, for example, key components of mentorship programs associated with successful outcomes (e.g., career satisfaction, academic outputs) and best practices deemed important to mentorship success. The literature recommends including a clear definition of mentorship, strategies to support appropriate matching of mentors and mentees and socialization of junior faculty into academic culture. Education to support the development of mentorship skills for both mentees and mentors is also emphasized. Other identified factors include leadership/institutional support and valuing the importance of mentorship [13–15]. We also consulted other departments within the Faculty of Medicine, known to have more formal faculty mentorship programs to learn about specific issues and the content that they included in the planning or evaluations of their programs.

### Survey

To encourage input across multiple sites all faculty members (*N* = 927) were invited to participate in a cross-sectional faculty-wide anonymous online survey. The structured 57-item survey was modelled, with permission, on previously described surveys in the literature of academic medicine [6, 9] (Supplement 1) and a survey conducted by the Department of Medicine, and initially developed by co-authors (MJE, EL, CH, JT, JW) who had experience in mentorship and/ or survey development. The survey was subsequently revised based on expert feedback (NK, SV, PS, KL, SD, IS, SS).

The survey was disseminated centrally by email including the survey link, with 5 reminders. All faculty were encouraged to participate and received a description/ purpose of the study and information that participation would remain anonymous. Announcements were also provided in the Department’s weekly newsletter. Email invites and encouragement also occurred directly through leadership at each academic hospital/ clinical setting. In addition, the Chair and Vice- Chair of Equity and Mentorship (MJE) for the Department encouraged participation, linking the announcement to the Department’s strategic plan and emphasizing input to inform creation of a new mentorship program.

Mentorship for the survey was defined as “a long-term relationship between two people where an accomplished individual with more experience, knowledge and connections takes a personal interest in helping to guide and develop a junior and more inexperienced person.”

Most items consisted of a 4 or 5-point Likert-type scale. Survey domains included professional characteristics, perceptions and attitudes about mentorship, perceptions of the departmental environment, including experiences of equity and professionalism, perceptions of key components relevant for the design of a mentorship program and topics of interest related to faculty development. Following questions on professional characteristics, survey questions asked participants about their access to mentorship (as a mentor/ mentee) and current or past experiences with mentorship, including perceived inequities or areas of disadvantage related to scholarly activities or opportunities.

Participants who identified as having a mentor were asked to rate the overall satisfaction with the mentoring received and the helpfulness of their mentor in relation to ten specific activities (e.g., reviewing scientific work, assistance in writing grants, opportunities for career advancement, advocacy with department leadership, introductions to individuals who could influence professional advancement, and advice on a variety of issues, such as academic promotion, as a clinician, researcher or teacher). These activities were rated on a 5-point scale (0 = *Poor*, 1 = *Fair*, 2 = *Good*, 3 = *Very Good*, and 4 = *Excellent*). A mentorship score was created by averaging the results from the responded questions. Participants indicating that they did not have a mentor were asked to rate the importance of mentoring in each of the ten specific activities on a 5-point rating scale (0 = *Not important* to 4 = *Very Important*).

The next section explored areas of perceived disadvantages. Faculty were asked (1 = *never*, 2 = *rarely*, 3 = *sometimes*, 4 = *frequently*, 5 = *always)* if they felt disadvantaged on issues relating to (1) ownership of intellectual property, (2) authorship disputes, (3) experience of questionable scientific integrity concerning a colleague, and (4) not receiving first authorship on a publication when having done most of the work. We created a mean score by averaging the results from the responded questions. To explore experiences related to diversity, equity, and professionalism within the Department, respondents rated on a 5-point Likert scale, (0 = *Strongly disagree*,* to 5 = Strongly agree*) the extent to which they agreed or disagreed that the Department (1) encourages and embraces diversity, (2) treats members with dignity and respect, and (3) works to correct systematic disadvantage to ensure equitable opportunities for all members. A mean score was created by averaging the results from the responded items. Participants were also asked if they felt their work or career advancement was hampered by discrimination related to gender, race, ethnicity, religion, disability, or sexual orientation (see questionnaire for definition of terms). Once again, a mean score was created.

Next, participants were asked about the perceived importance of demographic and career-related variables in assisting a mentee to find a mentor, potential design elements for a mentorship program (e.g., key desired components; use of technology), and interest level related to topics for educational workshops or tools to support mentoring activities.

### Ethical review

The survey was conducted as part of a project to create an academic mentorship program and conceptualized as a quality improvement initiative for the Department. The University of Toronto Review board approved the work as a quality improvement project and did not require ethics review. Need for informed consent was waived by the Human Research Ethics Program, University of Toronto. Informed consent was not obtained from participants.

## Analysis

Descriptive statistics (means, SDs, percentages) were used to summarize findings on demographics and responses. Chi-square statistics were used to compare differences for categorical variables between groups. Six survey questions included the option of open-ended responses. Content analysis of open-ended questions was completed by MJE and a research assistant with thematic analysis used to identify themes within the data. This involved a systematic process of coding and categorizing the responses to uncover themes, underlying meanings or insights. Through iterative review and discussion, consensus was reached on the emergent themes ensuring rigor and reliability in our analysis. Themes were then presented and discussed to assess further agreement in meetings with co-authors.

## Results

Data were collected from 225 respondents, representing a response rate of 24%.

### Characteristics of sample

Table 1 shows demographic characteristics of respondents. Most were Caucasian (68%), married/living with a partner (78.2%), between 41 and 50 years old (36%) and born in Canada (59%). Just over one-half of the sample identified as women (53%).

### Professional characteristics

The majority of respondents (*n* = 212, 94%) had a primary appointment with the Department; 70 respondents (31%) were non-physician faculty (e.g. PhD scientists/ psychologists). In terms of academic rank, 25% were Lecturers, 45% Assistant professors, and 20% were at the Associate, Full or Emeritus professor levels. Most respondents held an MD only (*n* = 104, 47%), PhD only (*n* = 54, 24%) or MD and Master’s degree (*n* = 41, 18%). Just under one-half (*n* = 99, 45%) held appointments with a Research Institute. On average, participants were in the Department for 11.4 years (*SD* = 10.9) and within their current academic rank for 6.6 years (SD = 7.0). Respondents reported working a mean of 48 h per week (SD = 12.1), with most spending time in direct patient care services and/or in research activities. In relation to academic roles, 30.6% were clinician teachers, 26.5% were clinician investigators/ clinician scientists (e.g. >75% protected time for research) and 18.7% were research scientists or psychologists. Others held roles as education scholars (18.7%) or worked in Quality Improvement (1.4%). Sample characteristics were similar to the overall breakdown in the Department (Female (52%; (χ^2^ = 0.122, *p =* .7264); Caucasian (60%; (χ^2^ = 4.949, *p* = .4221); Rank: Lecturer (32%; (χ^2^ = 4.170, *p* < .041); Assistant Professor (41%; (χ^2^ = 1.173, *p* = .2788), Associate (14%; χ^2^ = 4.260, *p* = 039), and Full Professor (11%; χ^2^ = 22.517, *p* < .001).

**Table 1:** Demographic characteristics of the sample (*N* = 225)

	*n*	%
Gender Identity *		
Woman	120	53.3
Man	82	36.4
Gender non-conforming	2	0.9
Prefer not to answer	21	9.3
*Age*		
<=40	55	24.4
41–50	82	36.4
51–60	46	20.4
61–70	17	7.6
> 70	9	4.0
Prefer not to answer	11	7.1
*Marital Status*		
Single	14	6.2
Living with a partner	21	9.3
Married	155	68.9
Separated/divorced	11	4.9
Other	2	0.9
Prefer not to answer	22	9.8
*Dependent Care*		
Yes	123	54.7
No	86	38.2
Prefer not to answer	16	7.1
*Children under the age of 12 living with you*		
Yes	80	65.6
No	42	34.4
*Disability Status (* *n* * = 29)*		
Visible	2	6.9
Non-visible	25	86.2
Both	2	6.9
*Person of Colour or Member of Visible Minority*		
Yes	61	27.1
No	153	68.0
Prefer not to answer	11	4.9
Race/Ethnic Background *		
East Asian	18	8.0
South Asian	24	10.7
White/Caucasian	136	60.4
Other (Black, Indigenous, West Asian, Middle Eastern)	8	3.6
Prefer not to answer	39	17.3
*Sexual Orientation*		
Heterosexual	169	75.1
Gay/Lesbian	15	6.7
Other (bi-sexual, not sure/questioning)	4	1.8
Prefer not to answer	37	16.4
*Born in Canada*		
Yes	133	59.1
No	83	36.9
Prefer not to answer	9	4.0

### Mentorship experience

Approximately two-thirds of respondents reported having at least one mentor (*n* = 152, 68%). Of those who did not have a mentor, 65% (*n* = 46) indicated interest in having one. Just over one-half (*n* = 123; 56%) of those with mentors had a mentor at their current clinical/research site, 36% (*n* = 79) elsewhere in the Department, and 20% (*n* = 45), elsewhere at the University of Toronto. Among respondents with mentors, 31% (*n* = 49) reported their mentor as being the person to whom they report. 61% of participants reported having mentors of the same gender (*n* = 96), 40% of same ethnicity (*n* = 63) and 65% of the same race (*n* = 103).

For those without a mentor, more women than men indicated that they would like one (χ^2^ = 5.014, *p* = .025, Cramer’s V = 0.282). More men compared to women had a mentor of the same gender (χ^2^ = 18.67, *p* < .001, Cramer’s V = 0.368), and more women compared to men indicated having a mentor of the same race (χ^2^ = 4.929, *p* = .026, Cramer’s V = 0.189). In comparing Caucasians to other groups, more men compared to women had a mentor of the same ethnicity (χ^2^ = 14.227, *p* < .001, Cramer’s V = 0.341) and race (χ^2^ = 56.402, *p* < .001, Cramer’s V = 0.661).

### Perceptions of satisfaction with or importance of mentorship activities

The majority of participants with mentors were satisfied with the mentoring received; overall satisfaction mean rating was 4.3 (SD = 1.05) (range: 1 = very dissatisfied to 5 = very satisfied). In considering whether a mentoring culture existed in the Department, the overall rating was 3.09 (SD = 0.81; 1 = *Strongly disagree* to 5 = *Strongly agree*). Similar results were found with the mentoring culture at the respondent’s primary clinical/research site (*M* = 3.41, *SD* = 0.96; 1 = *Strongly disagree* to 5 = *Strongly agree*).

**Fig. 1:**
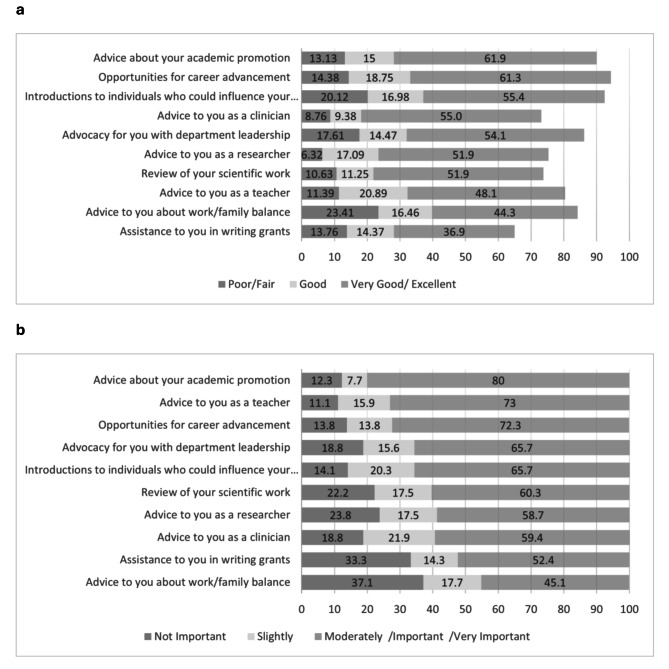
Overall helpfulness or importance with mentoring activities. **a**) For those with a mentor (percentage); **b**) For those without a mentor (percentage)

Figure 1a depicts perceived level of helpfulness in relation to specific mentoring activities among respondents with mentors. More than 50% of respondents rated advice about academic promotion, opportunities for career advancement, introduction to others, advice as a clinician, advocacy for department leadership, advice as a researcher, and review of scientific work as being *very good* or *excellent*. Participants without a mentor rated the importance of specific mentoring activities. 80% ranked advice about academic promotion, 73% advice as a teacher, 72% opportunities for career advancement and more than 60% reported advocacy with department leadership, introduction to others who could influence career and review of scientific work as being *moderately* to *very important*. (see Fig. 1b).

### Role as a mentor

Almost one-third of participants (*n* = 72, 32%) indicated serving as a mentor to an average of 3 faculty members (*SD* = 4.4); this did not vary as a function of gender (χ^2^ = 0.78, *p =* .702, Cramer’s V = 0.059), race (χ^2^ = 3.21, *p =* .073, Cramer’s V = 0.135), or academic rank (χ^2^ = 3.72, *p* = .445, Cramer’s V = 0.137). Mentors reported spending on average 2.6 h (SD = 2.8) in mentoring activities weekly. Only 26% (*n* = 19) of mentor respondents had received education or resources to support their role; however, 59% expressed interest in training. In terms of satisfaction, 86% of mentors reported that their mentee demonstrated appreciation of the mentorship provided and 76% indicated proactive behaviour was exhibited by their mentee (e.g., setting up meetings, preparation for meetings). Mentorship experience ratings among mentors did not vary by gender.

### Diversity and perceived inequities

In exploring perceptions of inequity or discrimination, 54% of participants indicated that they *somewhat agree* or *strongly agree* that the Department encourages and embraces diversity. 72% of respondents indicated that they *somewhat agree* or *strongly agree* that the Department has treated them with dignity and respect. Only 36% of respondents indicated that they *somewhat agree* or *strongly agree* that the Department works to correct systematic disadvantage to ensure equitable opportunities for all members of the Department.

**Fig. 2:**
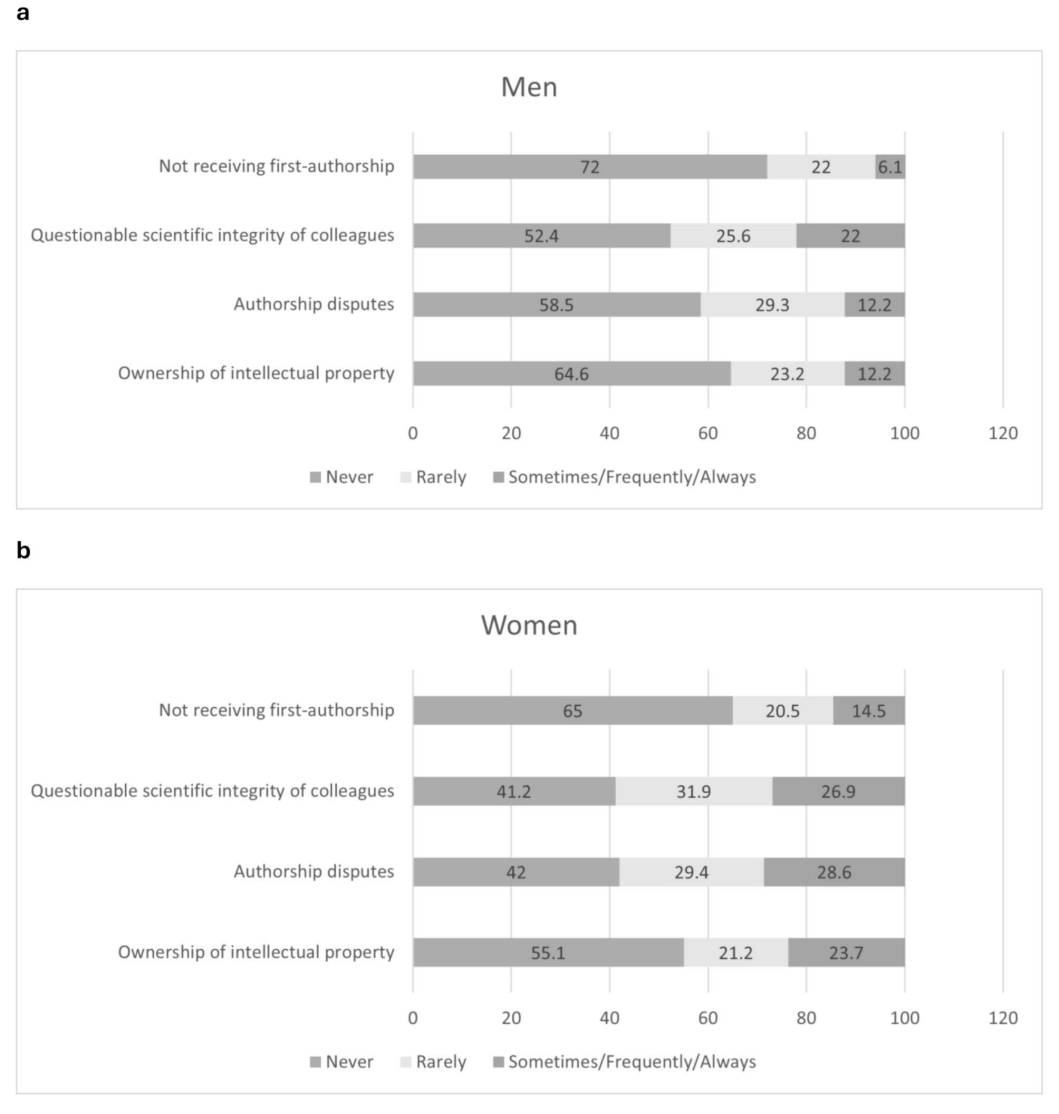
“Feeling Disadvantaged” by Gender (percentage)

Faculty members rated how often they felt disadvantaged in relation to ownership of intellectual property, authorship disputes, experience of questionable scientific integrity concerning a colleague, and in not receiving first authorship publication when having done most of the work. PhD faculty more often felt disadvantaged compared to MD faculty in not receiving first authorship (*M*_Non−MD_=1.64 ± 0.79 vs. M_MD_=1.36 ± 0.67; *t* = 2.51, *p* = .013); experiencing questionable scientific integrity concerning colleagues (*M*_Non−MD_ =2.01 ± 0.92 vs. M_MD_ =1.70 ± 0.81; *t* = 2.42 *p* = .017); and in experiencing authorship disputes (*M*_Non−MD_ =1.99 ± 0.91 vs. M_MD_ =1.66 ± 0.76; *t* = 2.63 *p* = .009). For both MD and PhD faculty, women compared to men were significantly more likely to experience authorship disputes in their academic work (χ^2^(2) = 8.67, *p* = .013). (see Fig. 2a and b).

Content analysis showed that some faculty felt a sense of “not belonging” or feeling disadvantaged (e.g. lacked access to mentorship or general information). International Medical Graduates and faculty who identified as being from a minority group expressed inequitable access to mentorship. Other themes related to site of work (smaller site), role (e.g. clinician teacher) or other demographic variables (e.g. age or gender) (see Table 2).

**Table 2:** Themes related to Status of Mentorship and factors to consider in Program Design

**Factors Associated With Not Having A Mentor** • No access/opportunity- “none were available”; “never felt supported by senior faculty”; “there is no one to be a mentor”; “not sure who to approach”; “didn’t seek it out” • Lack of Support for Mentorship- “no one interested in it”; “reached out and received unfavorable result”; “tried really hard”; • Feeling Disadvantaged -“being foreign graduate made it difficult” “overlooked because of who I am”; “overlooked due to being a woman”; “parental status is a thing they consider” “always nuanced” “not at a large academic hospital so we matter less”; “systematic disadvantages to do with site or place in the hierarchy, not just race or gender”; “age discrimination”; “being a mother”; “never feel I belong in academics and no one is really helping me figure it out”; • Barrier of Time- “nobody had time”; “everyone is busy with own career”; “too many things to do, mentorship falls off”; • Related to role-“I am clinician and do teaching but not offered mentorship”; “clinician investigators may not be guaranteed mentorship and may have to find own mentor”; • Unprofessionalism/Feelings of Discrimination- feelings of being less valued in academic career-“dismissed because I had an accent, so thought to be incompetent”; “lack of transparency regarding review process or renewal”; ”feeling powerless to express concern due to fear of further retaliation as I’m not from dominant culture”; “academic role and rank play a role, so there can feel like an academic undermining”; “ageism can play a role in who gets involved in mentorship”; “power differential” “don’t make waves if you have my background”.	
**Program Design Elements** ***Matching and Selection of Mentors*** • *Flexibility and mentee-driven mentee/ mentor matching strategy-* “doesn’t matter to me but for some race, ethnicity may matter”; “depends on what person wants”; “people may vary “; “I’m fine mentoring someone from different race or gender but it might matter to a mentee”; “gender, sexual orientation and parental status may be important for some”; “match to some extent on demographic variables”; • *Identify Mentors with Specific Qualities-* “confidentiality, trust and similar values important, including ethics”; “trust and empathy” “career level important for promotion”; “area of expertise or clinical interest important”; “fit and connection in the relationship more important than anything else”; “don’t force a match”; “don’t be restrictive”; “have adequate number of mentors to select from”; “have different site but in similar role” “post-website for mentees to see mentors”; “likely shouldn’t be your supervisor”. **Program Format** • *Caution not to over structure but need some structure-*“less structure the better”; “formal is good because it facilitates a culture of mentorship”; structure helps those who are quieter workers and don’t self-promote”; “needs to be driven by leadership and valued by department”; “some structure like goal setting, suggestion of regular meetings needed”; “on the fence about what needs to be documented based on mentee meetings and contact”; “need a website and structure”; “introduce it early in career”; “need structure otherwise people fall between cracks”; • *1:1 and Group Mentorship-* “I think groups would be good or some sort of peer forum”; ‘groups might foster cohesion”; “sharing of resources”; “1:1 important although a lot can happen with a group too but then people have to find convenient time”; • *Use of Technology****-****“*need face to face meetings not just virtual”; “technology can help save time; “mentorship website would be good to put tools on and announcements, bios”; “virtual could support relationship- building”; “technology is great, but you need a relationship”. • *Social /Networking Events-* “social event to support the program” “networking events or strategies are needed”; “help junior faculty meet others, we are very large department”; **Training/Tools to Support Mentors/ Mentees** • *Mentee/Mentor training-* “mentors can benefit from learning about how to be a good mentor”; “how to set goals”; “implicit bias training is needed”; “mentors need to work with variety of mentees, not just like themselves”; “how to foster connection”; “how to work with mentee from different gender or race”; “how to deal with conflict”; “are there ways to be a good mentee?”; “learning on what to document; how often to meet”; “mentorship during different phases of career”; “just overall communication would be good”; “How to sponsor your mentee”; • *Tools to support Activities* –“documenting or tracking goals might be helpful and to have some form”; “suggest use of a type of agreement in relation to meetings and mentoring role”; “virtual or online list serves or tools to support mentorship would be good”.	

### Design of mentorship program

**Fig. 3:**
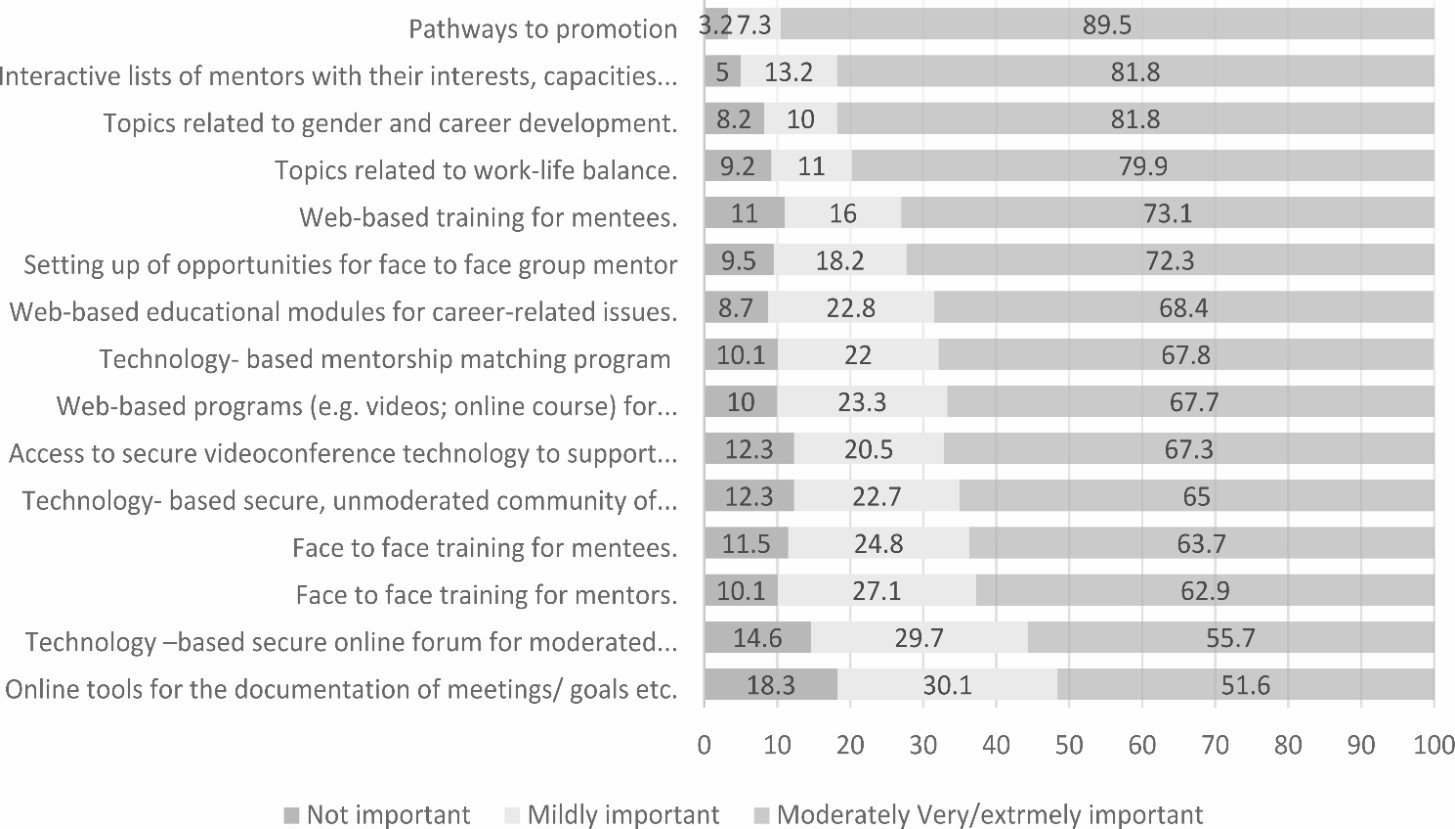
Perception of importance of specific components of mentorship program for the department of psychiatry (percentage)

Participants were invited to rank the importance among a list of components in designing a new mentorship program and for potential topics for education. Areas rated as *moderately/ very / extremely important* included: information on pathways to promotion (90%), having an interactive list of mentors with their interest/ expertise (82%), topics related to gender and career development (82%), topics related to work-life balance (80%), training for mentees (73%), opportunities for face to face mentorship groups (73%), web-based educational modules for career related issues (68%), a technology-based mentorship mentee/ mentor matching program (68%) and web-based program (videos, online course) (68%), technology- based unmoderated community of practice (65%), training for mentors (63%), and tools for documentation of meetings/ goals (52%) (see Fig. 3). Themes on suggested design elements from the content analysis are presented in Table 2 and include recommendations for program structure, the use of technology, mentee/ mentor matching strategy, training recommendations, and suggested mentor qualities.

In relation to variables important for assisting a mentee to find a mentor, almost two-thirds of participants (65%) deemed it was *very important or extremely important* to be matched on research/academic interests, followed by personality (59%). For social identity characteristics, 42% rated marital status, 33% rated sexual orientation, 25% rated parental status, 27% rated race (27%), 28% rated ethnicity, and 20% rated gender (20%) as being *not at all important*. Several respondents indicated that having flexibility in a matching strategy is important so that mentors can select based on preferences of demographics or areas of expertise. Relational fit, empathy, trust and maintenance of confidentiality were identified as key qualities that a mentor should have.

## Discussion

Findings from this needs assessment survey provide evidence to substantiate the initiation of a formal mentorship program for the Department. Although respondents indicated that to some extent a culture of mentorship existed there was room for improvement. More than one-third of respondents lacked access to a mentoring relationship and indicated a high interest in receiving mentoring. In the content analysis of open-ended questions, some faculty (e.g. International Medical Graduates; faculty identifying as a minority, women), indicated feelings of being isolated, “not fitting in”, lacking information on the academic and health system and access to a mentor. These challenges add to the burden of adaptation to the more practical aspects of an academic role, (e.g. teaching, research activities) and can impact academic productivity and career goals, resulting in career dissatisfaction and attrition [4, 7, 16]. Mentorship at the time of the survey was provided in an ad hoc manner. Informal systems of mentorship lack strategy for mentor/ mentee matching, clear expectations of mentor-mentee relationships, and institutional support for mentorship. This can be problematic as it is associated with inequitable access to mentorship. There is particularly strong evidence that women and minority-identifying groups experience disproportionately poor access to high-quality mentorship, with resultant negative impact on career development [17–19].

A significant number of respondents felt disadvantaged in relation to scholarly activities, particularly PhD faculty and women, and only 36% of respondents indicated that they “somewhat agree” or “strongly agree” that the Department works to correct disadvantage to ensure equitable opportunities for its members. These findings are consistent with the literature: groups who identify as marginalized often experience lack of opportunity, discrimination, and disadvantage [18–20]. PhD faculty are less than 20% of the total faculty in the Department and may feel challenged in their role identity or in feeling a sense of belonging and envisioning opportunities within a large department comprising mainly of physician faculty. A mentorship program targeting specific faculty groups may help address career dissatisfaction and feelings of inequity.

Among respondents receiving mentorship, satisfaction with mentoring was high and the value of mentoring was recognized among both mentors and mentees. Most mentees had a mentor at their local site (56%) or within the department (36%), with 31% indicating their mentor being in a supervisory role to them. The literature discourages a mentee choosing a mentor in a supervisory role, which can lead to the mentee seeking directive advice that may or may not be the best solution to a mentee’s situation or be influenced by a mentor’s competing interests [21].

Specific mentoring activities identified as being beneficial among faculty with or without mentors were consistent with the literature. As in other studies [1, 3, 5, 16] advice about promotion, career advancement, teaching, clinical skills, research skills and advocacy were identified as important benefits to mentorship. Newly hired faculty may come with some clinical expertise; however, most begin their academic careers with little formal clinical teaching experience. Among junior faculty in clinician investigator/scientist roles, gaining confidence in juggling clinical and academic demands and the need to learn skills in conducting research, as well as obtain information relevant for career advancement are crucial for success in their roles [3, 5]. A formal mentorship system, with access to more experienced and senior faculty can provide information and facilitate opportunities to network with other faculty members in similar roles, as well as assist in the socialization process and support career opportunities. Psychosocial supportive functions, such as mentor encouragement can foster the development of perseverance and resilience, characteristics needed to thrive in academia [4, 5, 21].

Overall, mentors reported feeling valued through expressed appreciation and feedback provided by their mentees. This finding is consistent with the literature showing that mentors gain career satisfaction and benefits through recognition in their role [21, 22]. However, few mentors had received formal workshops or tools, and the majority were interested in receiving education to support their role. Women showed greater interest in faculty development, similar to findings of others [23].

Qualitative themes indicated that mentoring education is important to ensure that mentors are approachable, interested in mentoring junior faculty, and remain accessible (e.g. agree to provide required time). Prior studies highlighted the benefits of mentor professional development and resources and have recommended incentives, such as awards to recognize mentors’ roles [5, 10]. Education on alignment of goals, best practices (e.g. importance of regular meetings), how to give feedback by mentors/ mentees within their relationship, and awareness of how to initiate and cultivate a mentorship relationship to mentee/ mentor’s best advantage are areas where skills can be developed [5, 21].

While the majority of respondents indicated that a formal program has advantages over informal mentoring activities, they varied in perspectives as to whether a formal program should include documentation, assigned mentors, or prescribed meeting frequency. Some respondents expressed caution around “over-formalizing” a program and underscored that any documentation or structure place utmost importance on the need to maintain confidentiality. Respondents suggested that an ideal mentor is one who maintains trust, empathy, confidentiality, and is aligned in an academic role or area of expertise. Personality was ranked more highly than gender, race and ethnicity. However, both quantitative survey rankings and content analysis showed that for some faculty having a mentor of similar race/ ethnicity was important.

Maintaining flexibility in the strategy supporting a mentee to choose their mentor, preferably from a menu of options based on preferences (including race, ethnicity, gender orientation or area of expertise) was recommended. This finding is consistent with the literature as formally assigned mentors can be a barrier to uptake or associated with less satisfaction with mentorship [1, 21]. Given the demographics of our department, we may have fewer opportunities to assist junior faculty mentees from racialized groups to obtain mentors with similar backgrounds. However, studies have shown that what is most important is regular access to a mentor, the sharing of expertise, and for a mentor differing in background from their mentee to have an understanding of how social identity may play a role in a mentee’s career [2]. Mentors can develop skills in providing a safe, caring environment with enhanced awareness of their own biases/ assumptions and how they can play a role in a mentee /mentor relationship [2].

This study describes the experiences of a cross-sectional cohort of faculty, however, there are limitations to note. While all full-time members in the Department were invited to participate, the response rate was 24%. We did not track nor collect information on faculty members who chose not to complete the anonymous survey. Thus, there is the potential for response bias as these results may reflect those with either poor or excellent experiences of mentoring or other factors that may have biased faculty members towards completing the survey. A contributing factor may also have been related to the survey occurring during the pandemic. The majority of faculty members hold clinical positions and would have been experiencing additional pressures and demands that may have affected interest level or the time available to participate in the survey.

Response rates for physician surveys are often lower than optimal; the rate in this study is similar to others surveying physicians [24]. A recent meta-analysis [25] discussed response rates and while authors note ideal responses, they concluded that there is no evidence suggesting that an 80% or higher response rate is an optimum response rate. They highlighted studies where samples were reliable even with a 5–10% response rate with large samples and found evidence that for surveys with a smaller sample size (e.g. less than 500) a response rate of 20–25% can be considered with “fairly confident estimates”. Our survey sample appeared to be representative of our faculty in terms of rank, position, gender, percentage who were Caucasian and level of education and we believe provides a fair representation of current mentoring practice.

While we collected a great deal of detailed information in the survey, there are content areas we did not inquire about that may be important related to barriers or facilitators to mentorship. For example, did having a sponsor or feeling disadvantaged play a role in further seeking out mentorship? How often were respondents meeting with a mentor/senior faculty member? Did experiences with mentorship as a student/ resident play a role in seeking out (or not) a mentor as a junior faculty?

### Application of study findings to create a new mentorship program

Findings from the study have informed the design and implementation of a more formal program described in detail elsewhere (in press). Recommendations in the literature and our study suggest key features of mentorship programs: a framework with a vision, defined values, and dedicated resources. Dedicated resources support an overall manager (0.5 FTE) who co-ordinates and implements the program. Participants in our study emphasized the use of technology both in terms of supporting meetings between mentors/ mentees, as well as for organizing training presentations, tools and other resources (e.g. storing and disseminating video recordings of presentations). We are incorporating an online platform to support the program in our plans.

Consistent with recommendations from participants and the literature we have included a core component of the program design to consist of a 1:1 mentorship (primary mentor/mentee) model; all newly hired faculty members, as well as current faculty without a mentor will be offered a mentor. In terms of recruiting mentors, while we do not have the available resources for salary support, we have invited mid and senior faculty who have a strong interest and experience in mentorship and who have agreed to provide the required time for activities.

Results from our study have informed an online matching program to support mentees to obtain a mentor based on their personal preferences (e.g. gender, race, ethnicity, area of expertise, role, etc.). As we recruit mentors, they complete a profile that includes their area of expertise, social demographic variables and academic roles. The online tool uses an algorithm to assign weights to social demographic factors and the more traditional variables of academic role and expertise to identify potential mentors. Each mentee faculty member is provided with up to 3 potential matched mentors based on their personal preferences and asked to explore mutual interests to select a primary mentor. Tools are provided via the online platform to support the 1:1 mentorship and include a mentee/mentor contract, an online template for tracking goals and progress and relevant videos on best practices. Educational workshops providing information on best practices (mentor/ mentee meeting frequency of at least twice monthly, empirical evidence supporting mentorship, tips for mentees to support the relationship) are provided at orientation for new faculty and for mentors. In addition, workshops related to DEI (diversity, equity, inclusion) and mentorship to support the mentor/ mentee relationship are offered.

The 1:1 mentorship component is further supported by several peer mentor led mentorship groups open to all faculty members. The mentorship groups help develop mentee knowledge and skills and provide psychosocial support relevant for specific academic roles or social identities. They also provide additional support to the primary mentor who may not possess all relevant expertise, or who may be of a different social background to that of their mentee. Participants in our study identified networking as important, in addition to skill building. For example, some faculty in specific roles (International Medical Graduates (IMGs) or who identified as diverse or marginalized, felt isolated. The multiple mentorship group options provide mentees with opportunities to suit their specific needs. The structure, process, and content of each mentorship group self-evolves. Each group is co-led by a peer mid/senior and a junior faculty member. Based on input from the needs assessment, we offer seven distinct mentorship groups that meet several times per year: (i) Clinician Scientists, (ii) Clinician Teachers, (iii) PhD Faculty, (iv) International Medical Graduates (IMGs), (v) Underrepresented Gender and Women (Uplifting Women, Underrepresented Genders and Diversity in Academia or “UPWURD”), with two subgroups: (vi) Racialized Women and (vii) LGBTQ2S+. The mentorship groups supporting faculty who self-identify as marginalized aim to provide a safe peer environment for added psychosocial support and networking.

Choi et al. [10] recommend efforts towards sustainability include built-in incentives. With this in mind, the mentorship program is linked to faculty re-appointment and annual reviews. This step involves faculty mentees indicating whether or not they are involved in the mentorship program and asking about the need for additional support in professional development. This process helps to identify mentees who have not successfully obtained a mentor or who are experiencing challenges in continuing with a mentor/mentee relationship, as well as systemic barriers or inequities among faculty. The program also provides opportunities for mentorship awards and recommends that junior and mid-career faculty include descriptions of mentorship activities in their dossiers when pursuing academic promotion. An evaluation plan, guided by a logic model (see Supplement 2), includes qualitative and quantitative methods to evaluate the program’s implementation and outcomes.

In summary, supporting academic faculty in their career development is important in facilitating career success and satisfaction. Our needs assessment survey showed that mentorship is valued and provided substantive evidence that a change was needed. A more structured mentorship program is an important strategy to increase the likelihood that equity and quality occur within mentorship activities. While some faculty cautioned around “over formalizing a program”, a more formalized program to address gaps in access and to encourage all junior faculty to develop a mentoring relationship aims to support roles and opportunities for skill-building and networking. We have built in flexibility to support mentees’ preferences, and the group component helps to address specific “needs” identified by faculty. Our program implementation plans incorporate a continuous quality improvement approach, so that any required iterative changes can be made along the way. An implementation science approach will also help us understand the depth and breadth of mentoring activities offered, strengths, and the challenges when a program is made more readily available to all faculty.

## Electronic supplementary material

Below is the link to the electronic supplementary material.

Supplementary Material 1

Supplementary Material 2

## Data Availability

The datasets used and/or analysed during the current study are available from the corresponding author on reasonable request. Survey is provided in supplement.
