# Supplementary material for: Identifying the mentorship needs among faculty in a large department of psychiatry- support for the creation of a formal mentorship program
Source: BMC Med Educ. 2025 Jan 11;25:47. doi: 10.1186/s12909-024-06629-y (PMC11724559; doi:10.1186/s12909-024-06629-y)
Supplement: Supplementary file 1 — Supplementary Material 1 [file 12909_2024_6629_MOESM1_ESM.docx]

Supplement 2: Survey Items and Interview Guide

**Needs Assessment Faculty Survey**

**SECTION 1: PROFESSIONAL CHARACTERISTICS**

1. Is your primary appointment in the Department of Psychiatry?

☐ Yes ☐ No

2. Is your appointment ‘status only’?

☐ Yes ☐ No ☐ Not sure

3. Please indicate what degrees you hold. Please check all that apply.

☐ MD

☐ PhD

☐ Other doctorate

☐ MSc

☐ MSW

☐ Other______________________________________

4. In what year were you first appointed to the Department of Psychiatry, Faculty of Medicine?

_________________(YYYY)

1. At what rank were you first appointed to the Department of Psychiatry, Faculty of Medicine?

☐ Lecturer

☐ Assistant professor

☐ Associate Professor

☐ Professor

6. Were you previously a faculty member at another University? ☐ Yes ☐ No

If yes, at what rank? _________________________________.

7. Please indicate your current academic rank in the Department of Psychiatry, Faculty of Medicine Rank (Select ONE response only)

☐ Lecturer

☐ Assistant professor

☐ Associate Professor

☐ Full Professor

☐ Emeritus

☐ Prefer not to answer

☐ Other (please specify)

1. In what year were you appointed to your **current rank** in the Department of Psychiatry, Faculty of Medicine?

_______________(YYYY)

1. Primary Hospital (select ONE response only-the site you identify with most).

☐ ***de-identified***

1. Do you belong to a Research Institute? ☐Yes ☐ No
2. Primary University Division (Choose one)

☐ Consultation / Liaison Psychiatry

☐ Psychotherapy, Humanities and Psychosocial Interventions

☐ Child and Youth Mental Health

☐ Adult Psychiatry and Health Systems

☐ Equity, Gender and Population

☐ Forensic Psychiatry

☐ Geriatric Psychiatry

☐ Neurosciences and Clinical Translation

1. Academic Position Description

☐ Clinician Administrator

☐ Clinician Educator

☐ Clinician Investigator

☐ Clinician- Quality Improvement

☐ Clinician Scientist

☐ Clinician Teacher

☐ Research Scientist

☐ Psychologist

☐ Prefer Not to answer

☐ Other ___________________________________________

1. Please indicate which *promotion track* you are currently on (or think you will be on): (if you believe you are on more than one promotion track, please check more than one box):

☐ Creative Professional Activity

☐ Research

☐ Teaching

☐ Education Scholarship

☐ Uncertain

1. Are you currently in a Departmental leadership position?

☐ No

☐ Yes (please check all that apply)

☐ Psychiatry-in Chief

☐ Department Division Lead

☐ Department Executive

☐ Promotions Committee

☐ Research Chair

☐ Other: ________________

**Distribution of professional time**

1. How many hours do you work in a typical work week? _____
2. What percentage (%) of these hours do you spend on average in each of the following professional activities? (Please enter 0 if you don’t engage in an activity)
   1. In direct patient care services: __(%)
   2. In all teaching activities __(%)

*(instruction to students, residents, interns and fellows in clinical lab and classroom settings)*

- 1. In all research activities __(%)

(in time spent writing proposals, papers, and reports, time spent planning and executing projects, and any laboratory time).

- 1. In administrative activities: __(%)

*(include clinical and academic administration, faculty and departmental meetings, and committees)*

- 1. In professional activities outside your institution __(%)
     *(Include consulting to outside groups, companies and agencies, professional society activities, lecturing to outside groups, employment in professionally related companies, and travel associated with the above)*

1. To what degree are you concerned about COVID-19’s current impact on your academic productivity?

☐ To a great extent ☐ To some extent ☐ Very Little ☐ Not at all

1. To what degree are you concerned about COVID-19’s impact on your academic career development?

☐ To a great extent ☐ To some extent ☐ Very Little ☐ Not at all

**Mentorship**

**The next group of questions pertain to the area of mentorship and will assist us in the development of a mentorship program. Please answer the questions within the context that the pandemic has been resolved.**

*A mentorship is a* ***long term relationship*** *between two people where an accomplished individual with more experience, knowledge, and connections takes a personal interest in helping to guide and develop a junior and more inexperienced person. Thus, the mentor is able to pass along what they have learned to a more junior individual (e.g. mentor may serve as a career role model and who advises and guides and promotes a mentee’s career or training).*

**Recognizing that people often have more than one mentor, we will be asking you to answer the following questions about your mentorship experience across all mentors.**

1. Do you have one or more persons who you consider to be a mentor (i.e., a long term relationship as described above) ?

☐ Yes

If yes, how many mentors do you feel you have? _____

☐ No

1. Why or why not? _________________________
2. Would you like a mentor? ☐ Yes ☐ No
3. Regarding all of your mentors, please check all that apply: (check n/a if no mentor)

I have mentors:

| At my site | Elsewhere in Department of Psychiatry | Elsewhere at ***de-identified*** | Outside of ***de-identified*** | N/A |
| --- | --- | --- | --- | --- |
| ☐ | ☐ | ☐ | ☐ | ☐  Skip to question 26 |

1. If you have one or more mentors, please choose a person to answer the following questions about your ‘primary’ mentor.

From your perspective, is he/she:

a. the person to whom you report? ☐ Yes ☐ No

b. the same gender as you? ☐ Yes ☐ No

c. the same ethnicity as you? ☐ Yes ☐ No

d. the same race as you? ☐ Yes ☐ No

1. Thinking of your other mentors, are any of them:

a. n/a (no other mentors) ☐

b. the person to whom you report? ☐ Yes ☐ No

c. the same gender as you? ☐ Yes ☐ No

d. the same ethnicity as you? ☐ Yes ☐ No

e. the same race as you? ☐ Yes ☐ No

1. Please rate your overall satisfaction with the mentoring that you receive from your mentor(s) with regard to:

(Check ONE response only for each row)

☐ N/A
☐ Very dissatisfied
☐ Somewhat dissatisfied
☐ Neutral
☐ Somewhat satisfied
☐ Very satisfied

1. In your experience, how would you rate the helpfulness of your mentor(s) overall with the following activities:

Poor Fair Good Very Good Excellent N/A

a. Review of your scientific work ☐ ☐ ☐ ☐ ☐ ☐

b. Assistance to you in writing grants ☐ ☐ ☐ ☐ ☐ ☐

c. Advice about your academic promotion ☐ ☐ ☐ ☐ ☐ ☐

d. Advice to you as a clinician ☐ ☐ ☐ ☐ ☐ ☐

e. Advice to you as a researcher ☐ ☐ ☐ ☐ ☐ ☐

f. Advice to you as a teacher ☐ ☐ ☐ ☐ ☐ ☐

g. Advice to you about work/family balance ☐ ☐ ☐ ☐ ☐ ☐

h. Opportunities for career advancement. ☐ ☐ ☐ ☐ ☐ ☐

i. Introductions to individuals who could influence your professional advancement.

☐ ☐ ☐ ☐ ☐ ☐

j. Advocacy for you with department leadership

☐ ☐ ☐ ☐ ☐ ☐

**Skip to Question 27**

1. **If no mentor**, please rate how important mentoring in the following areas would be to you?

Not Slightly Moder- Important Very ately Important

a. Review of your scientific work ☐ ☐ ☐ ☐ ☐

b. Assistance to you in writing grants ☐ ☐ ☐ ☐ ☐

c. Advice about your academic promotion ☐ ☐ ☐ ☐ ☐

d. Advice to you as a clinician ☐ ☐ ☐ ☐ ☐

e. Advice to you as a researcher ☐ ☐ ☐ ☐ ☐

f. Advice to you as a teacher ☐ ☐ ☐ ☐ ☐

g. Advice to you about work/family balance ☐ ☐ ☐ ☐ ☐

h. Opportunities for career advancement. ☐ ☐ ☐ ☐ ☐

i. Introductions to individuals who could influence your professional advancement

☐ ☐ ☐ ☐ ☐

j. Advocacy for you with department leadership

☐ ☐ ☐ ☐ ☐

1. To what extent do you agree the following are present within **Department of Psychiatry,** ***de-identified***?

|  | Strongly disagree | Disagree | Neither agree/nor disagree | Agree | Strongly agree |
| --- | --- | --- | --- | --- | --- |
| Environment that promotes a culture of mentorship |  |  |  |  |  |
| Environment that promotes feedback and sharing of information |  |  |  |  |  |
| Environment that promotes visibility |  |  |  |  |  |
| Environment that provides actionable advice and guidance |  |  |  |  |  |
| Knowledge of available department information |  |  |  |  |  |
| Knowledge of how to access departmental resources |  |  |  |  |  |
| Someone to turn to in case of difficulty |  |  |  |  |  |

1. To what extent do you agree the following are present at your Primary site (i.e., **hospital or research site**)?

|  | Strongly disagree | Disagree | Neither agree/nor disagree | Agree | Strongly agree |
| --- | --- | --- | --- | --- | --- |
| Environment that promotes a culture of mentorship |  |  |  |  |  |
| Environment that promotes feedback and sharing of information |  |  |  |  |  |
| Environment that promotes visibility |  |  |  |  |  |
| Environment that provides actionable advice and guidance |  |  |  |  |  |
| Knowledge of available department information |  |  |  |  |  |
| Knowledge of how to access departmental resources |  |  |  |  |  |
| Someone to turn to in case of difficulty |  |  |  |  |  |

1. In general, during your career, how often have you felt disadvantaged due to the issues of:
   1. Ownership of intellectual property

☐ Never

☐ Rarely

☐ Sometimes

☐ Frequently

☐ Always

b. Authorship disputes

☐ Never

☐ Rarely

☐ Sometimes

☐ Frequently

☐ Always

1. Questionable scientific integrity of colleagues

☐ Never

☐ Rarely

☐ Sometimes

☐ Frequently

☐ Always

1. Working for scientist/ supervisor leading much of work but not receiving first author publication

☐ Never

☐ Rarely

☐ Sometimes

☐ Frequently

☐ Always

**Mentorship Experience as a MENTOR**

*Definitional reminder: A mentorship is a* ***long term relationship*** *between two people where an accomplished individual with more experience, knowledge, and connections takes a personal interest in helping to guide and develop a junior and more inexperienced person. Thus, the mentor is able to pass along what they have learned to a more junior individual (e.g. mentor may serve as a career role model and who advises and guides and promotes a mentee’s career or training).*

28.

a. Are you currently a mentor (as defined above) to other Faculty members ***de-identified***?

☐ Yes

☐ No

b. If no, are you interested in being a mentor?

**} Go to Question 34**

☐ Yes

☐ No

1. How many people do you currently mentor? _____
2. In a typical work week, how many hours do you spend in mentorship activities? ___(Hours)

*(Include all time spent advising others on issues related to their professional/career development and academic activities, including reviewing of their talks, research proposals, and manuscripts)****.***

1. a. Have you received training or resources to support this role? ☐ Yes ☐ No

b. Are you interested in receiving training to support this role? ☐ Yes ☐ No

1. Please rate your satisfaction with how your mentorship is valued (recognized) at the following:
   1. ***de-identified***, Department of Psychiatry

☐ N/A
☐ Very dissatisfied
☐ Somewhat dissatisfied
☐ Neutral
☐ Somewhat satisfied
☐ Very satisfied

- 1. Your primary hospital or research institute site

☐ N/A
☐ Very dissatisfied
☐ Somewhat dissatisfied
☐ Neutral
☐ Somewhat satisfied
☐ Very satisfied

1. Please rate your satisfaction with how your mentorship is in relation to:

|  | Strongly dissatisfied | Somewhat dissatisfied | Neutral | Somewhat satisfied | Strongly satisfied | N/A |
| --- | --- | --- | --- | --- | --- | --- |
| proactive behavior by mentee (e.g. setting up meetings, reaching out, preparation for meetings) |  |  |  |  |  |  |
| mentee providing feedback on your mentorship |  |  |  |  |  |  |
| mentee demonstrating appreciation for your mentorship |  |  |  |  |  |  |
| mentee informing others in the department of your mentorship activities |  |  |  |  |  |  |

**Design of Mentorship Program**

1. Please rate the extent of importance of the following approaches in our development of a mentorship program for the Department of Psychiatry. We are also looking at ways that technology could be leveraged at the Department of Psychiatry level to optimize mentorship capacity. Please rate the following ideas in terms of how useful you believe the initiatives would be:

|  | Not important | Mildly important | Moderately important | Very important | Extremely important |
| --- | --- | --- | --- | --- | --- |
| **Identification of potential mentors** | | | | | |
| Assistance in matching:   1. Interactive lists of mentors with their interests and capacities on a secure website. |  |  |  |  |  |
| 1. Technology- based mentorship matching program (e.g. e-referral service where a person inputs interests/ needs and then gets centrally triaged to potentially appropriate mentor. |  |  |  |  |  |
| **Support of 1:1 Mentorship meetings** | | | | | |
| 1. Accessibility to zoom technology for 1:1 meeting between mentor/mentee across sites. |  |  |  |  |  |
| **Group mentorship** | | | | | |
| 1. Setting up of opportunities for face-to-face group mentorship. |  |  |  |  |  |
| 1. Access to secure videoconference technology to support group mentorship across sites. |  |  |  |  |  |
| 1. Technology- based secure, **unmoderated** community of practice for sharing of information/ discussion. |  |  |  |  |  |
| 1. Technology –based secure online forum for **moderated** discussion boards. |  |  |  |  |  |
| Web-based educational modules for career-related issues. |  |  |  |  |  |
| Online tools for the **documentation** of meetings/ goals etc. |  |  |  |  |  |
| **Training for mentors on mentoring** | | | | | |
| 1. Web-based programs (e.g. videos; online course) for mentors |  |  |  |  |  |
| 1. Face to face training for mentors |  |  |  |  |  |
| **Training for mentees** | | | | | |
| 1. Web-based programs for mentees |  |  |  |  |  |
| 1. Face to face training for mentees |  |  |  |  |  |
| **Specific Mentorship topics that could be delivered in groups:** | | | | | |
| 1. Gender and career development |  |  |  |  |  |
| 1. Work-life balance. |  |  |  |  |  |
| 1. Pathways to promotion |  |  |  |  |  |
| 1. **Other topics? _________________________________________________________________________________________________________________________________________________________________________________________________________________________________________________________** | | | | | |

1. Regardless of how you feel about matching, if the Department creates a process to help people find a mentor, in your opinion, how important would it be for that process to match on:

|  | Not at all important | Not very important | Somewhat important | Very important | Extremely important |
| --- | --- | --- | --- | --- | --- |
| Gender |  |  |  |  |  |
| Ethnicity |  |  |  |  |  |
| Race |  |  |  |  |  |
| Sexual Orientation |  |  |  |  |  |
| Marital status |  |  |  |  |  |
| Parental status |  |  |  |  |  |
| Personality |  |  |  |  |  |
| Research interests |  |  |  |  |  |
| Administrative interests |  |  |  |  |  |
| Speciality/-subspeciality |  |  |  |  |  |
| Other: Please specify _________ |  |  |  |  |  |
|  |  |  |  |  |  |

1. Please share any other ideas you may have in relation to the development of a mentorship program or in the use of technology to support mentorship:

**SECTION 2: EQUITY, DIVERSITY, AND PROFESSIONALISM**

1. To what extent do you agree or disagree that the Department of Psychiatry encourages and embraces diversity?

☐ Strongly disagree

☐ Somewhat disagree

☐ Neither agree or disagree

☐ Somewhat agree

☐ Strongly agree

1. To what extent do you agree or disagree that the Department of Psychiatry treats you with dignity and respect?

☐ Strongly disagree

☐ Somewhat disagree

☐ Neither agree or disagree

☐ Somewhat agree

☐ Strongly agree

1. To what extent do you agree or disagree that the Department of Psychiatry works to correct systematic disadvantage to ensure equitable opportunities for all members of the Department?

☐ Strongly disagree

☐ Somewhat disagree

☐ Neither agree or disagree

☐ Somewhat agree

☐ Strongly agree

1. Please rate the extent to which you believe your work or career advancement in the Department of Psychiatry has been hampered by discrimination related to…

To a great extent To some extent Very Little Not at all

- 1. Gender ☐ ☐ ☐ ☐
  2. Race ☐ ☐ ☐ ☐
  3. Ethnicity ☐ ☐ ☐ ☐
  4. Religion ☐ ☐ ☐ ☐
  5. Sexual orientation☐ ☐ ☐ ☐
  6. Disability ☐ ☐ ☐ ☐
  7. Other ________________________________________

1. a. Have you **witnessed** or **personally experienced** what you perceive to be “unprofessionalism” (for example, issues of disrespect, harassment, abuse, microaggression*, and discrimination) by other colleagues, towards others (for example, trainees, other researchers within the Department/Division or otherwise, interprofessional colleagues) or towards yourself within the last three years?

*(*note: Microaggression is defined as brief and commonplace daily verbal, behavioural, or environmental indignities, whether intentional or unintentional, that communicate hostile, derogatory, or negative prejudicial slights and insults toward any group, particularly culturally marginalized groups)*

Witnessed unprofessionalism ☐ Yes ☐ No

Personally experienced unprofessionalism ☐ Yes ☐ No

**If no to both, skip to Question 42**

1. What types or form(s) of unprofessionalism did these events (collectively) include? (Check all that apply)

☐ Disrespect

☐ Abuse

☐ Microaggression

☐ Discrimination

☐ Other (please specify) __________________________________

1. Were any of the following specific ‘groups” or individuals targeted in any of the above event(s)? Check all that apply:

☐ Gender/sex

☐ Race/Ethnicity

☐ Sexual orientation

☐ Other (please specify)

☐ No specific group targeted.

1. Did you take action/report **any** of the events that you witnessed/experienced at that time?

☐ Yes ☐ No

If no, why not? ________________________________________________

1. Please rate your confidence that you can take action to address unprofessionalism without concern for reprisal.

☐ Very doubtful
☐ Somewhat doubtful
☐ Neither confident/nor doubtful
☐ Somewhat confident
☐ Very confident

1. Have you had any formal training on unconscious bias with the last three years?

☐ Yes ☐ No

1. Have you had any formal training on equity, diversity and/or inclusion within the last three years?

☐ Yes ☐ No

**SECTION 3: PERSONAL DEMOGRAPHICS**

1. Age (years)

☐ ≤30

☐31-40

☐ 41-50

☐ 51-60

☐ 61-60

☐71-80

☐ >80

☐ Prefer not to answer

1. What is your present gender identity? (Please check ONE only)

☐ Woman (cisgender)

☐ Woman (transgender)

☐ Man (cisgender)

☐ Man (transgender)

☐ Gender Non-Conforming

☐ Gender Fluid

☐ Non-binary

☐ Two-spirit

☐ Prefer not to answer

☐ Other (please specify) ________________

Personal Relationships/ Dependents

1. What is your marital status?

☐ Single

☐ Living with a partner

☐ Married

☐ Separated/Divorced

☐ Widowed

☐ Prefer not to answer

☐ Other (please specify) ________________

1. Do you care for any dependents (this may include a child, parent, other family member, relative with a disability etc.)?

☐ Yes

☐ No

☐ Prefer not to answer

1. Do you have any children age 12 years or younger living with you either full-time or part-time (this includes biological children, adopted children, step-children, foster children, grandchildren, or other children for whom you are responsible)?

☐ Yes

**} Go to Question 50**

☐ No

☐ Prefer not to answer

1. How many children are living with you that are:
   1. age 12 or younger ___
   2. age 13 to 16 ________

**Disability Status**

*A person with a disability is someone who has a long-term or recurring physical, mental, sensory, psychiatric or learning disability and considers oneself to be disadvantaged by reason of that disability, or believes that society is likely to consider them to be disadvantaged by reason of that disability. A person with a disability may also be someone whose functional limitations owing to their disability have been accommodated in their environment.*

1. Whether or not it affects your day-to-day life, are you a person with a disability?

☐ Yes

} **Go to Question 53**

☐ No

☐ Not sure

☐ Prefer not to answer

1. Is your disability ….

☐ Visible

☐ Non-visible

☐ Both

☐ Prefer not to answer

1. What type of disability do you have? (Select All that apply)

☐ Physical, functional and/or mobility disability (e.g. Arthritis, paraplegia, cerebral palsy, muscular dystrophy, spinal cord injuries, spina bifida)

☐ Blind and/or low vision

☐ Deaf, deafened and /or hard of hearing

☐ Speech disability (e.g. stuttering)

☐ Chronic medical condition (e.g. diabetes, chronic pain, HIV/AIDS, systemic exertion intolerance disease, kidney disease, seizures disorders)

☐ Developmental disability (e.g. Asperger’s Syndrome, Autism, Fetal Alcohol Spectrum disorders)

☐ Learning disability (e.g. dyslexia)

☐ Psychiatric disability and/or mental health disability (e.g. bipolar disorder, obsessive compulsive disorder)

☐ Prefer not to answer

☐ Other (Please specify) ________________

**Racial/Ethnic Identity**

*In Canada, a person of colour or a member of a visible minority group is defined as someone (other than Indigenous Person) who self-identifies as non-white in colour, regardless of birthplace or citizenship. Members of ethnic or national groups (such as Portuguese, Italian, Greek, etc) are not considered to be racially visible unless they also meet the criteria above.*

1. Do you consider yourself to be a person of colour or member of a visible minority in Canada?

(Select ONE response only).

☐ Yes

☐ No

☐ Prefer not to answer

*Indigenous is a term used to describe the original inhabitants of Canada and their descendants. Indigenous people in Canada include First Nations, Inuit and* Métis *people. An Indigenous person may be a treaty status or a non-status, registered or non-registered Indian.*

1. Are you an Indigenous person in Canada? (If yes, please check One only)

☐ Not Indigenous (please go to next question)

☐ First Nations

☐ Métis

☐ Inuit

☐ Urban Indigenous

☐ Prefer not to answer

1. Which of the following best describes your race/ethnic background? (Select ALL responses that apply)

☐ Indigenous (First Nations, Inuit or Métis person from any country)
☐ Black
☐ East Asian (Chinese, Japanese, Korean, etc.)
☐ South Asian (Indian, Pakistani, Sri Lankan, East Indian from Guyana, etc.)
☐ Southeast Asian (Filipino, Cambodian, Indonesian, Laotian, Vietnamese, Thai, etc.)
☐ West Asian (Iranian, Iraqi, Persian, etc.)
☐ Central Asian (Kazakh, Afghan, Tajik, etc.)
☐ Middle Eastern
☐ White / Caucasian
☐ Prefer not to answer

1. What is your sexual orientation? (Select ONE response only)

☐ Asexual/Non-sexual

☐ Bisexual

☐ Gay

☐ Heterosexual (“straight”)

☐ Lesbian

☐ Two-spirited

☐ Pan-Sexual

☐ Not sure/Questioning

☐ Prefer not to answer

☐ Other (please specify) _______________

1. Were you born in Canada?

☐ Yes

☐ No

If no, at what age did you immigrate to Canada? ______

And from what country? ______________

This is the end of the Survey.

Thank you for your assistance.
