# Supplementary figures and images for: Identifying the mentorship needs among faculty in a large department of psychiatry- support for the creation of a formal mentorship program
Source: BMC Med Educ. 2025 Jan 11;25:47. doi: 10.1186/s12909-024-06629-y (PMC11724559; doi:10.1186/s12909-024-06629-y)

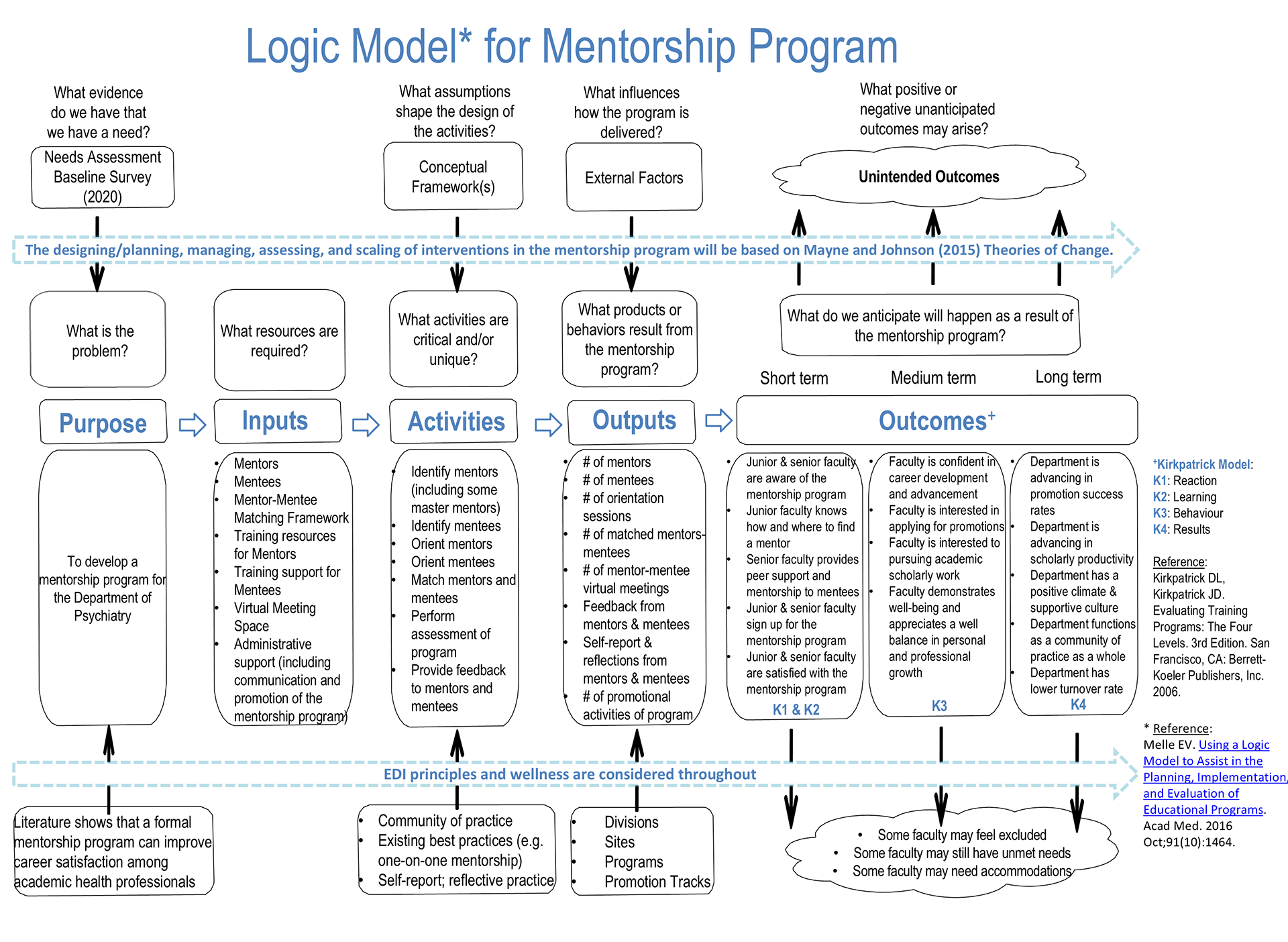

Supplement: Supplementary file 2 — Supplementary Material 2 [file 12909_2024_6629_MOESM2_ESM.png]
